# Digital pathology evaluation of complement C4d component deposition in the kidney allograft biopsies is a useful tool to improve reproducibility of the scoring

**DOI:** 10.1186/1746-1596-6-S1-S5

**Published:** 2011-03-30

**Authors:** Ernesta Brazdziute, Arvydas Laurinavicius

**Affiliations:** 1 National Centre of Pathology and Vilnius University Faculty of Medicine, Vilnius, Lithuania

## Abstract

Complement C4d component deposition in kidney allograft biopsies is an established marker of antibody-mediated rejection. In the Banff 07 classification of renal allograft pathology, semi-quantitative evaluation of the proportion of C4d-positive peritubular capilaries (PTC) is used. We aimed to explore the potential of digital pathology tools to obtain quantitative and reproducible measure of C4d deposition in the renal allograft tissue.

34 routine kidney allograft biopsies immunohistochemically stained for C4d were included in the study and were evaluated by a qualified pathologist twice, recording an approximate percentage of positive PTC and glomerular area. The same slides were scanned by Aperio ScanScope scanner. Two layers of annotations were created: layer of glomeruli and the remaining non-glomerular area. Image analysis was performed with Aperio Positive Pixel Count algorithm to quantify the proportion of C4d-positive pixels in the area analysed. The percentage of positive (defined as 2+ and 3+) pixels in glomeruli and non-glomerular area was obtained and compared to the percentage of C4d-positive PTC and C4d-positive area of glomeruli recorded by the pathologist.

The correlation of digital and manual C4d-positive area scoring in glomeruli was very high (r= 0.89, p<0.0001), while the correlation for non-glomerular (digital) and PTC (manual) area was moderate (r=0.60, p<0.001). The correlation between digital and manual evaulation of C4d in non-glomerular area after exclusion of C4d-positive arterioles from analysis did not improve substantially (r = 0.59, p < 0.001). Reproducibility of digital and manual results was evaluated. For C4d deposition in PTC, agreement between the first and the second digital C4d evaluation (after re-drawing annotations) was perfect (κ=0.96, CI 0.91÷1.00) while agreement between two subsequent manual C4d scorings was substantial (κ = 0.67, CI 0.47 ÷ 0.88). Similarly, for C4d deposition in the glomeruli, agreement of digital evaluation was perfect (κ=1) while for manual scorings it was substantial (κ = 0.76, CI 0.64 ÷ 0.88).

Digital evaluation of C4d deposition in allograft kidney correlates with pathologist‘s scoring and exceeds the latter in reproducibilty. Therefore, it provides a useful tool to control for intraobserver and interobserver variability and may serve as quality assurance measure for allograft pathology diagnosis and research.

## Background

Renal biopsy is the gold standard for acute rejection in renal transplant recipients where both humoral and cellular rejection mechanisms play the role. Acute humoral rejection may be associated with the appearance of anti-donor specific antibodies. In renal biopsy, complement split product C4d deposition in the peritubular capillaries (PTC) is considered to be a marker of antibody-mediated anti-donor humoral response [1,2].

In 2007, Banff classification of renal allograft pathology was updated with C4d deposition scoring scheme thus increasing the importance of robust and reproducible measurement of C4d deposition [3]. Several studies have demonstrated a remarkable inter-observer variability of histological grading of the kidney allograft pathology [4,5]. Interobserver reproducibility for the score of C4d expression in PTC was moderate (k=0.57-0.63) while intraobserver reproducibility was substantial (k=0.68–0.83) [6]. Furthermore, Furness et al. have shown that reproducibility of Banff classification throughout Europe was low, revealing that international variation is even greater than inter-observer variation within small groups of pathologists working in the same institution or country.

Digital pathology is an emerging technology that provides quantitative tools to improve measurement and reproducibility of grading, including that of renal allograft rejection. Successful application of digital techniques has been shown in renal allografts for interstitial fibrosis [7] and tubulitis [8] scoring.

To our knowledge, the potential of digital technologies to evaluate C4d deposition in renal allograft has not been explored. Therefore, we aimed to investigate concordance and reproducibility of the measurements produced by a pathologist and Aperio Positive Pixel Count algorithm.

## Materials and methods

This study included 34 kidney allograft core needle biopsies from 32 patients.

**Figure 1:**
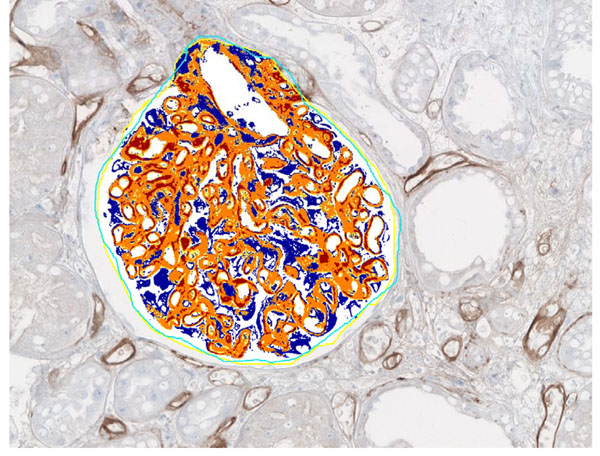
Automated image analysis in glomerular tissue. Manually selected glomeruli were analysed with Aperio Positive Pixel Count algorithm. Different colors indicate the intensity level of C4d positive pixels (red – 3+, orange - 2+, yellow – 1+ ).

**Figure 2:**
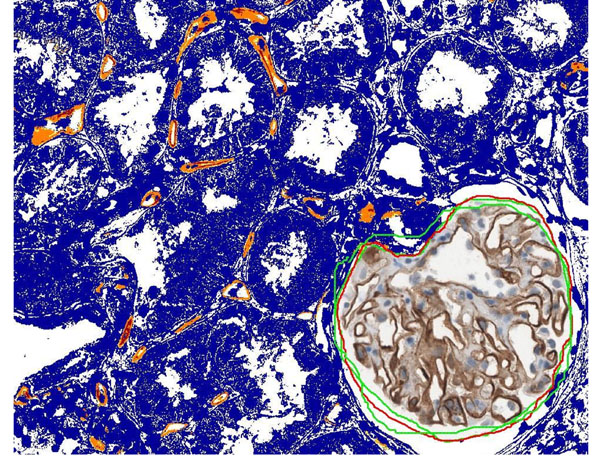
Automated image analysis in non-glomerular area. Glomeruli were excluded from this annotation layer. C4d positive peritubular capillaries are marked with orange (2+ intensity pixels) or red (3+ intensity pixels) colors.

Sections (3 micrometer-thick) of formalin-fixed paraffin-embedded renal tissue were stained with rabbit anti-human C4d polyclonal antibody (polyclonal antibody, Spring Biosience). Glass slides with biopsy tissue were viewed and evaluated twice by pathologist (AL) scoring the percentage of C4d-positive peritubular capillaries (PTC) and the percentage of C4d-positive area of the glomeruli. The same 34 glass slides were scanned with Aperio ScanScope scanner using “Faint” parameters in order to obtain a better quality of virtual slides. The slides were viewed and annotated with Aperio ImageScope program by another investigator (EB). The first layer of each virtual slide annotations was created manually selecting glomeruli-only area (all glomeruli on the section) (Fig. 1), the second layer included all remaining area of renal tissue (except glomeruli and areas of significant fibrosis, Fig. 2). Both layers were separately analyzed with automated image analysis algorithm Aperio Positive Pixel Count v9 using default set of parameters. To test the reproducibility of automated image analysis, it was repeated after re-creating the annotations and running the algorithm with default parameters.

An algorithm output provided a number of 1+, 2+ and 3+ intensity positive pixels and the number of total pixels in each annotated layer (glomeruli and non-glomerular tissue). Total number of pixels consisted of positive and negative pixels excluding white area of the virtual slides (i.e., the center of tubules, vacuoles, Bowman’s capsule space). Since 1+ pixel areas were mostly picking-up weak background staining of the tubules and other structures but not C4d deposition in the capillaries, only 2+ and 3+ areas were regarded as C4d-positive for further analysis. To compare pathologist’s scores with Aperio algorithm results, the percentage of 2+ and 3+ pixels was calculated.

Pathologist’s scores and automated image analysis results were compared using Pearson’s correlation and weighted kappa concordance statistics. To measure inter-observer variability between manual PTC and automated non-glomerular tissue scoring, scores were divided: a) manual and automated scores into 5 equal intervals; b) manual scores into three groups according to Banff 07 classification: 0–10% (C4d0-1), 11–50% (C4d2), and 51–100% (C4d3) while automatic scores of non–glomerular and glomerular C4d deposition were divided into three intervals 0–1, 1.1 – 2.6, >2.6% and 0–5, 5.1–15.9, >16 %, respectively (“3 thirds”); c) manual scores divided the same like in b), but automated scores divided into 3 intervals with the same proportion of cases like in manual scoring Banff intervals (“automated Banff”); d) manual scores divided into six groups: 0, 1, 2-10, 11-30, 31-60 and 61-100% while automated scores were divided into 6 groups with the same number of cases like in each manual score interval. Analysis was performed with SAS 9.2 statistical package.

## Results

### C4d deposition in glomeruli

The positivity values obtained by automated analysis in all virtual slides ranged from 0.11 to 73.21%, with a mean of 18.96 ± 21.18%. The pathologist scored C4d deposition in glomeruli estimating approximate proportion of C4d-positive area in all glomerular capillaries observed. The scores ranged from 0 to 100% with a mean of 43.40 ± 40.66%.

**Figure 3:**
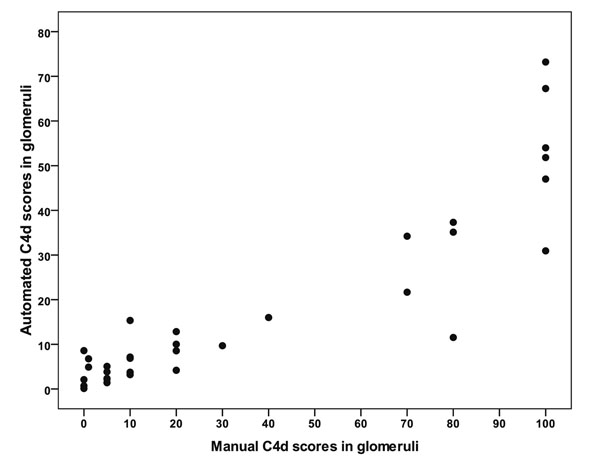
A plot of manual and automated evaluation of C4d deposition in glomeruli.

Correlation between the pathologist’s and digital score was strong (r=0.89, p<0.0001). The values are plotted on the Fig. 3. To avoid the impact of asymmetrical distribution of the values, we also performed Pearson’s correlation with logarithmic values (r=0.82, p<0.0001).

### C4d deposition in non-glomerular area

The positivity values obtained by automated analysis in non-glomerular tissue area averaged at 3.02±3.01 (range 0.23-13.57%). One case with an outlier value of C4d positivity at 13.57 was observed: a review of this case revealed cellular rejection (Banff class IA) with an extensive C4d deposition in the interstitium and tubular basement membranes. This case was excluded from further analysis. After exclusion of this case, average positivity values were 2.57 ± 2.25 (range 0.23–8.77%). The pathologist’s score of C4d-positive PTC ranged from 0 to 100%, the mean was 28.55 ± 31.36%.

**Figure 4:**
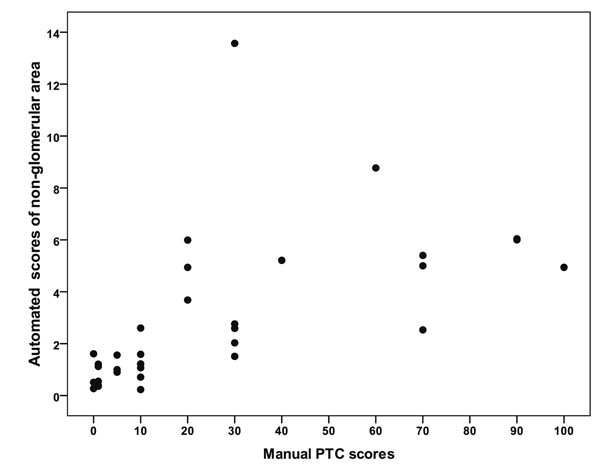
A plot of manual and automated evaluation of C4d in the non–glomerular area (one outlier case (Banff class IA) at 14% automated score can be noted).

Moderate (0.60, p<0.001) correlation was observed between the manual and automated scores, plotted on the Fig. 4. Exclusion of one case mentioned above, increased this correlation up to 0.77 (p<0.001). To avoid the impact of asymmetrical distribution of the values, we performed additional tests that confirmed the relation: Wilcoxon signed ranks test (p<0.001) and Pearson’s correlation with logarithmic values (r=0.79, p<0.0001).

To test potential impact of occasional C4d–positivity in the arterioles on the automated score, we performed additional analyses of the non-glomerular area after manual annotation excluding the arterioles from the automated analysis. The average scores slightly but not significantly decreased (3.02 to 2.81) and the correlation with the manual score did not change substantially - 0.59 (p< 0.001).

### Inter- and intraobserver variability

Intraobserver agreement for manual glomerular area scores ranged from 0.76-0.87 for various grouping of cases while intraobserver‘s agreement for automated glomerular area scores was perfect (k=1). Kappa values for interobserver agreement for manual and automated glomerular scoring (subdivided into various intervals, not shown) ranged from 0.68 to 0.79.

Kappa values for various scoring intervals of the non-glomerular area are presented in the Table 1. While intraobserver concordance of manual evaluation ranged at kappa values of 0.67 to 0.71, intraobserver concordance of the automated scoring was excellent (k=0.93÷1). Remarkably, manual-automated interobserver agreement (kappa values in the range of 0.50 to 0.71) was comparable to that of manual intraobserver variability.

**Table 1:** Inter- and intraobserver‘s variability for non-glomerular area scores

	Dividing scores into 5 equal intervals		Manual Banff and automated "3 thirds" intervals		Manual Banff and automated "Banff Aperio" intervals		Manual and automated scores divided into 6 intervals	
	
	Intra- var.	Inter- var.	Intra- var.	Inter- var.	Intra- var.	Inter- var.	Intra- var.	Inter- var.
Manual	0.70		0.71		0.71		0.67	
		0.51		0.50		0.71		0.62
Automated	0.93		1		1		0.96	

## Discussion

Many studies reveal rather low inter- (k=0.57) and intrapathologist‘s (k=0.68) variability for semi-quantitative evaluation of pathological changes, especially when pathologist‘s work in different centers and countries. In fact, this reveals the need of more objective and reproducible measurement not only in clinical practice but also for research and clinical studies. We tested a hypothesis if automated image analysis tool – Aperio Positive Pixel Count algorithm – is comparable to the pathologist‘s scoring and could add benefits of automated and reproducible measurement. As our data showed, automated scoring strongly correlated with manual scores for the glomerular and moderately for the non-glomerular C4d deposition. Interobserver agreement (k=0.50 - 0.71) did not differ substantially from intrapathologist‘s agreement (k=0.67- 0.71) while the reproducibility of automated scoring was much better (k=0.93- 1) than that of manual scoring. Although automated measurement produces a different parameter (proportion of positive pixels in the whole non-glomerular area) from the manual scoring (proportion of positive PTC), they both aim to measure relative quantity of C4d in the renal tissue. We assumed that in cases without signifficant fibrosis, atrophy or inflammation, the number of peritubular capillaries is more or less the same from case to case, therefore the measurement of positive pixel area should correlate the manual measurement of C4d according to the Banff07 classification. Since the concordance measures revealed similar manual-automated interobserver and manual intraobserver variability at the level of kappa of 0.7, automated analysis of C4d could be a useful tool for quality assurance and intercenter studies with the advantage of better intraobserver agreement.

## Conclusion

Digital evaluation of C4d deposition in allograft kidney correlates with pathologist‘s scoring and exceeds the latter in reproducibilty. Therefore, it provides a useful tool to control for intraobserver and interobserver variability and may serve as quality assurance measure for allograft pathology diagnosis and research.

## Competing interests

The authors declare that they have no competing interests.

## Authors‘ contributions

Both authors contributes equally.
